# Facilitating change processes in group-based behaviour change interventions in rural African contexts: practical lessons from Ghana

**DOI:** 10.1186/s13033-023-00571-0

**Published:** 2023-02-06

**Authors:** Richard Appiah

**Affiliations:** 1 College of Health Sciences, University of Ghana, Accra, Ghana; 2 Center for African Studies, Harvard University, Cambridge, MA USA; 3 Department of Psychology, University of Johannesburg, Johannesburg, South Africa

**Keywords:** Facilitation strategies, Group-based behaviour change interventions, Mental health interventions, Behaviour change process, Ghana, Rural Africa

## Abstract

Evidence from implementation research suggests that group-based behaviour change interventions (GBCIs) can encourage the development of peer support, promote psychosocial skills, and facilitate collaborative therapeutic relationships. However, although the mechanisms of action that mediate the behaviour change process have been extensively described in other settings, less is known about the implementation strategies and contextual factors that actuate the reported behaviour changes among programme participants in Ghana and sub-Saharan Africa, more generally. We draw on insights from the literature and field experiences from designing, implementing, and evaluating GBCIs across several rural and peri-urban communities in Ghana to discuss a range of theoretical, methodological, and contextual factors that facilitate the behaviour change process in programme participants. We offer suggestions to guide researchers to envision and manage potential challenges with the programme development and implementation processes. We propose that intervention programmes designed to facilitate health behaviour change in the defined context should (i) have a context-relevant focus, (ii) be coherent and well-structured, (iii) have explicit techniques to facilitate inter-personal and intra-personal change processes, (iv) include appropriate mechanisms to monitor and assess the progress of the interventional sessions; and (v) be implemented by trained facilitators with a deep knowledge of the sociocultural values and norms of the target group and of the principles and theories underlying the intervention programme. We envisage that these insights could serve to guide the design, implementation, and evaluation of contextually-tailored and potentially effective GBCIs that align with the needs, capacities, and circumstances of the local population.

## Background

A wealth of empirical evidence from systematic reviews and meta-analyses suggests that group-based interventions are effective in supporting health improvements and behavioural changes across population groups and contexts [1–3]. The group-based approach permits the delivery of intervention sessions in a group format to a selected number of participants. This delivery strategy is time-efficient and cost-effective [4], facilitates the development of peer support, social networks, and collaborative therapeutic relationships [5], and carries longer lasting effects [4]. There has been a growing interest in applying the group-based approach to facilitate positive behavioural changes in the healthcare, community, and work settings—with promising outcomes [6, 7]. Beyond its primary function as a well-established implementation strategy to facilitate the delivery of intervention programme sessions simultaneously to selected individuals, the group-based intervention approach has been shown to actuate the therapeutic change process and to expedite the attainment of the intended behavioural changes [8]. More recently, a number of theories have been postulated to explain the mechanisms that underpin the change processes in group interventions [8, 9].

Although empirical research exists that describes the change processes in group-based behaviour change interventions (GBCIs) in other settings [1, 8], there is limited literature in Ghana, and sub-Saharan Africa more generally, that discusses the therapeutic factors that drive the behavioural changes that are reported in the literature. Identifying and characterising group-level change processes and providing a detailed analysis of what happens in groups could enhance our understanding of the mechanisms of action in strengths-based and health behaviour change interventions in the low-income African context, where mental health is heavily stigmatised and community-based mental health interventions are limited. Literature that discusses the design features and change processes that occur in groups as well as the strategies that can be utilised to facilitate these changes can guide researchers to design and implement more effective and context-appropriate behaviour change intervention programmes for the target population.

## Main texts

In this paper, we carefully reflect on, and draw insights from, the literature and experiences from designing, implementing, and evaluating the effects of three GBCIs for adult residents of rural and peri-urban communities in Ghana to discuss a range of theoretical, methodological, and contextual factors that instigate and facilitate the behaviour change processes in GBCIs. The three GBCIs, thus, the Escaping Poverty project [10], the Inspired Life Program [6, 11], and the Parental Nudges Project [12], involved developing and evaluating or adapting multicomponent strengths-based and positive behaviour change intervention modules, which were subsequently administered to about 2000 adults randomly recruited from 185 rural poor communities across eight regions of Ghana.

We discuss the facilitation strategies and contextual factors capable of potentiating the behaviour change processes, under two domains. The first, *pre-intervention considerations*, comprise an explication of the focus and scope of (health) behaviour change intervention programmes relevant to the defined context; the format and structure of the programme sessions; and the characteristics and skill sets of session facilitators required to bolster interest of participants and increase the interactivity of sessions. The second domain, *in-session considerations*, encompasses delivery strategies that can be adopted to facilitate inter-personal and intra-personal change processes, as well as mechanisms to monitor the progress of sessions. We conclude with a discussion of lessons learned from conducting GBCIs in Ghana and offer some practical recommendations to guide prospective researchers in the design and implementation of group-based health behaviour change interventions for the defined contexts.

### Pre-intervention considerations

#### Focus and scope of GBCI programmes

Health behaviour change intervention programmes are driven by particular goals, contents, and philosophies in achieving their purpose of promoting positive behavioural changes [13]. To maximise their intended benefits, it is imperative that GBCI programmes targeting residents of rural and socioeconomically-disadvantaged communities in sub-Saharan Africa are designed to impact multiple aspects of well-being and personal growth. In addition to its well-defined aims and outcomes, the overarching goal of the intervention programme can be multifocal—by targeting multiple domains of mental health and social skills or vocational productivity, such as improving participants’ intra-personal feelings (e.g., sense of well-being and happiness), positive inter-personal skills (social networks and relationships), positive growth and functioning (vocational productivity), or build their resilience in a general sense. Unlike clinical populations who present with specific health deficits, individuals in most rural poor communities in sub-Saharan Africa are often constrained with a wide array of psychosocial (e.g., high levels of stressors; limited access to medical, educational, and social resources) and existential challenges (e.g., high susceptibility to diseases and poverty) in various degrees of complexity [11]. Researchers, from the study conceptualisation phase and throughout the research process, should endeavour to engage with the communities and local stakeholders in mutually respectful, trusting, and collaborative ways to identify the actual needs of the target group and set study objectives that align with the identified needs in order to increase interest and participation in the programme activities and maximise their impacts. For instance, although the research team set out with specific objectives for the ILP, a priori, the researchers followed the Medical Research Council’s framework for developing and evaluating complex interventions to randomly select a sample of community leaders and members from the target population to solicit views on the proposed programme content, structure of the sessions, and implementation strategy. This was necessary in order to ensure that the final intervention programme matches the needs, capacities, and circumstances of the target group. This formative phase of the intervention development also tasked participants to suggest revisions to refine the proposed programme [11, 14]. Generally, programme participants who set and evaluate self-identified goals are more motivated to achieve their goals [15].

Given the level of socioeconomic deprivation (and its potential adverse effects on residents in these settings), the inclusion of themes that advance the acquisition of socioeconomic skills may be an important component worthy of consideration for GBCIs or health promotion intervention programmes for participants residing in rural, socioeconomically-disadvantaged communities of sub-Saharan Africa. A growing body of evidence shows that intervention programmes that target the promotion of positive mental health and resilience also lead to improved economic outcomes, thus yielding a cycle of increasing returns [16]. Such studies have recommended the integration of components of socioeconomic skills training into mental health interventions. Research suggests that a multitude of factors contribute to mental health disorders, many of which are unlikely to be overcome by a single intervention [17]. These sorts of causal chains suggest that it is beyond the scope of any one intervention to address the root cause of poor mental health in low-income settings, which is intrinsically linked to economic productivity. For instance, a study in Ghana that examines the association between employment and psychological distress found that increased psychological distress was associated with increased likelihood of being unemployed, and further estimated that the lost productivity associated with psychological distress translates to approximately 7% of Ghana’s gross domestic product [18].

We surmise that existing evidence support, and unquestionably warrant, the expansion of the scope of mental health and behaviour change intervention programmes targeting low-income communities to include session themes that set out to teach socioeconomic skills such as goal setting, problem-solving, time-management, and financial literacy skills. In a study in Ghana that explores participants’ experiences, perceived benefits, and recommendations to improve a 10-session group-based multicomponent positive psychology programme [14], the majority of participants recommended that the programme be expanded to also include facets of physical health. We contend that this quest for a broader, holistic interventional package provides further evidence for a biopsychosocial approach to health and well-being that takes into consideration the physical and mental health aspects of an individual, which also essentially underscores the complementarities between mental and physical health [14, 19]. Overall, we recommend that GBCIs targeting the promotion of positive mental health in the socioeconomically-disadvantaged settings of Africa be complemented with some form of economic components (e.g., asset transfers) or socioeconomic skills training (e.g., goal setting, problem-solving, and relationship and communication skills) to facilitate participants’ uptake and application of the lessons and skills embedded in programmes.

#### Structure of programme sessions

Considering that the majority of individuals residing in rural poor communities have low educational attainments, who may also be unable to take notes at intervention programme sessions [11], interventional sessions targeting these population groups could be structured such that a large part of the session time is allocated to group discussions and activities—rather than long lectures and commentaries by session facilitators. To the extent that the majority of programme participants may tend to rely primarily on reflection and recollection of the discussions and activities at the sessions to help them to apply the knowledge and skills at a later time [14], it may be useful to apportion a significant proportion of the session time for group discussions or activities in breakout and plenary sessions to ensure that the key lessons of the sessions are well understood.

We note that the manner in which the sessions are structured can weigh significantly on the delivery, participation, and uptake of the intervention programme. The programme sessions for the ILP, for instance, were carefully structured in a manner that optimised interest, acceptance, and the potential impact of the programme. The initial part of the first session, for example, provided a framework to establish rapport with participants, introduced the main goals of the programme, set group norms and rules to regulate sessions, clarified the session agenda, and discussed the main theme for the session using contextually-relevant examples. The session also included two breakout sessions where participants further discussed key themes and practiced skills with their partners to gain mastery. In a plenary session that followed, facilitators reviewed key components of the session, discussed home assignments, and concluded the session with a brief discussion of the theme for the next session. Participants thereafter took turns to describe their experience or views about the session. All the subsequent sessions followed a similar pattern. While we do not suggest this structure as a gold standard, we would recommend that the initial session of GBCIs designed for the defined population be used to reinforce the aim and purpose of the intervention programme, motivate participants to commit to attend all sessions in order to maximise the benefits from the programme, and to address participants’ questions and concerns. Further, we urge researchers to organise separate sessions for each gender group when working in the more patriarchal African community settings, where women are likely to be less articulate and interactive when in the midst of men [20, 21].

#### Session facilitators

The development and implementation of GBCIs often involve substantial expenditure on field staff and logistics that need careful consideration. Nonetheless, a growing trend is to recruit and train non-expert local volunteers to lead the delivery of the sessions. Yet, because session facilitators are the key drivers of the programme implementation, it is important that they have requisite knowledge of the theories and principles underlying the intervention programme. Given that the sessions would likely be held in the local dialect of participants, it is important that researchers engage facilitators who are fluent in the dialect of the target population (or be native speakers themselves, where possible); are familiar with the cultural norms and customs of the people; are friendly, approachable, and courteous; and where possible, should have previous experience of delivering programme sessions in similar contexts. We note that the characteristics and personal attributes of session facilitators substantially affect the quality of delivery, interactivity, and fidelity of the group, especially because the intervention sessions are typically designed to be highly interactive and participatory in order for participants to gain understanding and mastery of skills in-session.

Further, a successful implementation of GBCIs in the rural, more collectivistic and highly synergistic sub-Saharan African setting would require that facilitators have a deep understanding of, and adhere to, the cultural norms and values of the people [14, 20, 21]. Of note, programme participants in rural poor communities may be somewhat sensitive to facilitators’ comments and gestures, especially when facilitators are not native speakers of the local dialect. We urge session facilitators to be courteous and respectful in their expressions and engagements with participants in order to build rapport and nurture positive relationships with participants and excite their interest in the programme. We note that a practical strategy to enhance acceptance, participation, and overall progress of the programme in the defined context is for facilitators to accede to, foster, and demonstrate a heightened sense of *epistemological humility*, by considering the views of participants and engaging with them as peers and co-creators of knowledge, rather than take an all-knowing professorship posture.

### In-session considerations

#### Facilitating inter-personal change processes

The claim that group-based intervention programmes enhance the development of peer support, psychosocial skills, and collaborative therapeutic relationships may be attributed, in part, to the interpersonal changes that are reported by programme participants [22]. As postulated by the social learning theory [23] and the African ontology and traditional African thought [24], people learn from observing and interacting with others. The process by which mutual engagements and self-disclosure in group-based learning leads to the behaviour change and skill development need to be emphasised to programme participants to encourage participation.

Canvassing and encouraging member support among group members remains one of the most efficient ways of enabling inter-personal change processes [1, 8]. We would strongly recommend that facilitators provide each participant an opportunity to share their views on each subtheme under discussion, citing context-relevant example situations of how the lessons and skills can be applied. Participants should be directed to take turns to speak, ask questions for clarification, and suggest context-appropriate examples to support their views. Such level of interactivity provides a forum for participants to learn from the narratives of the facilitators and from the views and examples shared by group members. Breakout sessions can also be used as avenues to facilitate inter-personal changes. In this regard, session facilitators can pair up participants and task them to generate ideas, practice a skill for proficiency, or brainstorm and suggest solutions to a problem scenario. Subsequently, each pair discusses their findings or views with the group in a plenary session. Breakout sessions can also create opportunity for facilitators to provide feedback to each individual participant and observe their progress.

We note a few sociocultural issues that deserve critical attention when implementing health behaviour change intervention programmes in the rural, low literate contexts of sub-Saharan Africa. Firstly, the GBCIs may be the first of its kind for some participants who may be uncertain of what would be expected of them. Participants should be reminded periodically that the group meetings and discussions are novel and that there are no specific behavioural expectations of participants. Secondly, it is possible that some programme participants may be anxious or unsure whether their contributions or responses would be appropriate. Participants should be informed that the goal of the discussions is not to evaluate their contributions, but instead, to learn from the facilitators and each other. We note that some rural and peri-urban communities of Ghana are more patriarchal—and females and young male adults may be less assertive when in the midst of elderly males. We would encourage researchers to consider organising separate sessions for the gender groups where this applies, in order to encourage participation of female participants. The approaches to envision and manage these ethico-cultural issues in the Ghanaian context have been discussed elsewhere [20].

Although participants in GBCIs learn from the interactive group discussions and activities as much as from session facilitators’ expositions and commentaries [1, 8, 14], an important strategy—which is practicable and akin to the more collectivistically socially-oriented African communities—is to encourage participants to share the lessons and skills they learned at the sessions with family members and neighbours as part of the home assignments. We observed that participants achieved mastery over skills when they shared insights from each session with two or three non-participants, solicit their views on the topic, and provide feedback from these engagements with the group in the subsequent session. Further, facilitators can task participants to describe their personal experiences and aspects of their lives where the lessons from the session can be applied, before the close of each session.

#### Facilitating intra-personal change processes

The strategies to ensure the practicality of group intervention sessions in the rural Ghanaian context have been previously described [20]. Following an elaborate discussion of the aims and purpose of the intervention programme, participants can be supported to set personal goals and indicate their expectations of each session. After each topic is discussed and activities completed, facilitators can solicit feedback from each participant to assess their progress. Since GBCIs are largely interactive, which allows members to share their views and learn from each other [8], facilitators should encourage participants to cite context-relevant, personal, and practicable examples that members can easily understand and learn from. Each participant, at the end of each session, can also be tasked to recount the main lessons and skills they acquired from the session and how they can apply them to promote their well-being and personal growth. Together with session facilitators, participants should evaluate their progress towards achieving their personal goals at the end of each session. Facilitators can use information from the progress review to improve on the content and delivery of subsequent sessions, which is an inherent strategy to enhance both intra- and inter-personal change processes.

We found it expedient to also conduct in-session evaluation of participants’ understanding of the lessons and mastery of skills and their overall progress. We tasked each participant to briefly reflect on the main theme and key lessons of the session, demonstrate a specific set of skill and discuss when they can be applied, and describe aspects they find difficult to understand or gain mastery. The facilitators carefully note each participant’s strengths and weaknesses and offer praise and clarifications, as necessary. It needs to be emphasised to participants that there is need for continuous practice of the skills learned in each session in order to gain proficiency.

Because a considerable number of individuals in the defined context may be unable to read or write, facilitators should adopt practical and context-appropriate approaches to deliver the sessions in order to expedite participants’ understanding and retention of the information learned at the sessions. For instance, researchers utilised the empty glass jar demonstration in the Escaping Poverty project and Inspired Life Program study to teach goal-setting, problem-solving and time management skills. One of two empty jars of similar size and volume was filled with a quantity of moderate-sized pebbles, followed by smaller pebbles, sand, and water. Subsequently, the second jar was filled with the same quantity of items, but this time in a reverse order, thus: water, sand, smaller pebbles, and, the moderate-sized pebbles. Whereas the first jar was completely filled (with a small space to spare), the second could hardly accommodate all the moderate-sized pebbles. Participants were thereafter asked to reflect on the demonstration and decipher the symbolic meanings of the glass jars, pebbles, sand, and water, and how the lessons therein can be applied to manage time effectively, set personal and family goals, and solve personal and communal problems.

#### Monitoring the progress of sessions

It is possible—and should be expected—that each programme participant may encounter some challenges that could hinder their efforts to understand the lessons and skills embedded in a specific session. We would encourage facilitators to periodically evaluate participants’ understanding of the discussions during the breakout sessions, and task them to discuss aspects of the session they find difficult to comprehend or a skill they have yet to master. Participants who have clear understanding of the topic or have mastered a particular skill can be asked to volunteer to offer further support to members by leading the review or demonstrating the skill to colleagues in the group. We strongly encourage periodic breakout sessions to grant facilitators the opportunity to evaluate and provide individual-level feedback to each participant and to encourage and assist participants who need additional support to improve on a skill, while also commending those who have demonstrated mastery over these skills.

The strategies instituted by the research team to monitor and evaluate the process and progress of the intervention programme form a critical component of the intervention process [2, 8]. A trained session monitor can be tasked to sit at the back to observe the proceedings of the session. The role of the session monitor should be discussed with participants at the initial meeting. The session monitor makes no contributions or remarks at the sessions, but only draws the attention of the facilitators when an important section of the discussions has been skipped, or provide a general feedback to session facilitators at the end of the session. We found the following scoring criteria invaluable to the progress and success of the sessions, including whether: (i) facilitators are in prompt attendance and decently dressed (ii) the session themes and goals are well written on a flip chart and well positioned, and (iii) participants are seated in a semi-circular or circular pattern to allow the ease of making eye contacts with each other. The session monitor also scores the delivery and proceedings of the intervention sessions on a 10-item criteria, including whether: (i) facilitators are methodical with the session manual (ii) the pace or speed of the delivery is appropriate (iii) the session is interactive (iv) facilitators respond appropriately to participants’ questions (v) participants’ responses indicate understanding of concepts (vi) facilitators cheer up group slogans/motto to enliven session, as necessary (vii) facilitators collaborate and coordinate in the delivery of the session (viii) participants are offered adequate time to interact with each other and practice skills during breakout sessions (ix) there are any missed sessions with a group, and (x) there are well-articulated plans to organise missed sessions for participants and group. The monitor debriefs the facilitators at the end of each session and discuss their observations and aspects of the facilitation that need further improvements.

### Lessons learned and practical recommendations

We note that a number of theoretical, methodological, and sociocultural factors complot to influence the uptake and effectiveness of GBCIs in the defined contexts. Firstly, the community-based participatory research (CBPR) approach, which was adopted in all three (and a dozen other) intervention programmes—where participants sit together to brainstorm solutions and learn from each other, aligns with the fundamental concepts of African epistemology and the knowledge-based system of the African societies. In the African thought and culture, people are more interconnected and relational and define personhood in terms of a person’s association with others [25, 26]. In the highly collectivistic African settings, programme participants are likely to view their well-being and growth in relation to the progress and success of others, thus engendering a collective sense of responsibility and a high motivation to participate, which, in turn, could optimise the benefits for participants. Unlike the African cultural orientation where this level of synergy and collectivism may be explicitly expressed, Western cultures place more importance on individuality, autonomy, and independence of thought [20, 21, 25]. Secondly, although there is a large body of evidence that suggests that constructs and principles of positive psychology and cognitive-behavior models have been found effective in promoting positive behaviour changes across contexts [27–29], including the treatment of common mental disorders such as anxiety disorders and depression [30], their effectiveness as a promoter of psychological well-being in the general population has not yet been proven to the same extent in the low-income contexts of sub-Saharan Africa. Based on our findings [6, 11, 14] and field observations, we contend that the majority of the psychological constructs and principles used to formulate the Inspired Life Program, Escaping Poverty project, and Parental Nudges Project are applicable to the African context. We find that it is feasible and important to translate psychological theories into potentially effective, context-tailored behaviour change intervention modules to promote mental health in the low-income settings of Africa.

Thirdly, in addition to building resilience and positive mental health, the theories and concepts that underpinned the above-mentioned programmes also inherently promote values and virtues such as peace, happiness/satisfaction, helpfulness, collective responsibility, cooperation, interdependence, and reciprocal obligations, which are akin to the African cultural values [26, 31]. Another possible reason for the significant effects of the Inspired Life Program, for example, was that a homework assignment was given at the end of each session, which further tasked participants to discuss lessons or practice skills with non-participants and to solicit their views on a set of questions. Previous research evidence suggests that engaging in intentional activities could promote well-being and advance the application and integration of lessons and exercises into daily lifestyles [5, 32, 33].

For the majority of programme participants, the group sessions provided them the first opportunity and forum to interact with other members in a more formal, yet social milieu to discuss and brainstorm solutions to their personal and collective problems. We observed that our initial discussions on ‘ground rules’ were essential in setting the boundaries of engagement to guide the conduct of the sessions. Because the sessions encouraged participants to share their personal experiences with the group to help other members draw lessons from these narrations, it was important to emphasise on the need to keep these personal examples and reflections as confidential. We extended the discussions to also include the need for participants to commit to attending sessions and on time, to give others the opportunity to also contribute to the discussions, and to demonstrate more appropriate ways to express their disagreements to comments made by others. These discussions, we noted, engendered a high sense of decorum and also encouraged participants to reflect and share their personal experiences as practical examples for the discussions—which helped to place the lessons into perspectives.

Similar to other sociocultural contexts, a considerable number of Ghanaian (and African) communities are patriarchal, where cultural and customary norms require men, for the most part, to take lead in the decision-making process—especially at meetings where both gender groups are present. Although the contributions of women in these meetings carry almost equal weight as those of men (e.g., most communities have queen mothers who partner with the chief or king in decision-making), most female participants are more likely to support the contributions of their male counterparts, unless, of course, they starkly disagree. In order to boost interactivity of female participants, we would recommend that researchers organise separate sessions for the gender groups, particularly in the rural, highly patriarchal communities. We noted, consequently, that the exclusive sessions with the female groups were equally, if not more, interactive. Although it would be important to observe how both genders respond to each other’s views on the themes, it was not our objective to explore differences in opinions between the gender groups.

GBCIs are usually structured to commence with a welcome note from facilitators, where participants and facilitators exchange pleasantries [1, 8]. From the onset, facilitators should endeavour to facilitate positive group cohesion and interactivity. For instance, in the first (introductory) session, participants can be paired up and tasked to inquire about their partner’s name, marital status, occupation, preferred name to be called at sessions, one interesting thing about them, and other details that may be relevant for the meetings. Partners thereafter take turns to introduce each other to the group in a plenary session. Further, facilitators can initiate a discussion about rules of engagement for the meetings and lead participants to generate specific group rules and expectations. We found that initial discussions on the need to keep personal experiences shared at the sessions as confidential and the need for members to allow others to take their turns to share their views instilled decorum and encouraged members to share their views and experiences with the group at sessions, at their own volition—which maximised the benefits of shared learning. Group members can also be encouraged to choose a group motto or slogan, with a matching response, that is periodically cheered to enliven the sessions. We note that participants often choose words and phrases that represent strength, courage, unity, and resilience (usually akin to their cultural values) as their group slogans, or responses to them.

A central feature of CBPR is that researchers collaborate with community stakeholders in a mutually respectful and trusting manner and work together to conceptualise, implement, and evaluate research activities [34]. For most community-based implementation studies, there is need to organise logistics (including community resources) and participants to attend the programme sessions. However, it can be particularly difficult to organise participants to attend sessions on time, since a considerable number of them may not have access to telephones or reliable network connectivity. We found it valuable to confer with community leaders to identify, recruit, and train an individual to support the research team to identify and organise resources such as venues to hold the sessions, lead the team to borrow (or rent) and arrange tables and chairs for the sessions, as well as to visit participants in their homes (or sometimes nearby farms in remote rural villages) to remind them to attend the sessions. The last role of the community mobiliser is especially important because two separate interventional sessions were held on each day in different communities, requiring that sessions are organised within a specific agreed time. Since the community mobilizer becomes an invaluable member of the research team, a descent remuneration for their services should be agreed upon. The community mobilizer is different from the independent mediator—who is also recruited (usually with inputs from the community leaders) and trained to lead the research team into the households to introduce the research team and the study, help the team to address all concerns of prospective participants, and support the informed consent and recruitment processes.

## Conclusions

This paper provides the first insight into the facilitation strategies and contextual factors that can significantly weigh on the designing, implementation, and uptake of GBCIs in Ghana. Drawing on insights from the literature and previous field experiences, we highlight some theoretical, methodological, and contextual factors that can instigate and facilitate behaviour change processes in programme participants in the defined context. We envision that these insights may provide valuable information to prospective researchers in their design, implementation, and evaluation of context-appropriate health behaviour change intervention programmes in the non-urban, low-income settings of sub-Saharan Africa. We propose that GBCIs designed for participants in this context should be well structured with well-articulated techniques to facilitate inter-personal and intra-personal change processes, and should have well outlined mechanisms and measures to monitor and assess the proceedings of the programme sessions. While we encourage researchers to engage local volunteers as facilitators, it is important that they are adequately trained to gain the requisite knowledge of the principles and theories underlying the intervention programme, of the skills and art for the session delivery, as well as of the cultural norms and customs of the target group.

We agree with previous suggestions [29, 31] that GBCIs designed for the low-income, vulnerable population groups should essentially adopt a multicomponent approach that include sessions that target the reduction of negative affect, such as anxiety and depression, with others also focusing on promoting positive mental health, building resilience, and optimising positive experiences and strengths, as advocated in the third wave of positive psychology [35, 36]. Given recent findings that socioeconomic status and increased vocational productivity improve mental well-being [37, 38], we would urge researchers to also include programme components that focus on economic empowerment and life skills training when working to promote the mental health of vulnerable population groups. While much remains to be learned, community-based behaviour change intervention programmes hold much promise in attempts to improve the mental health of adult populations in resource-limited settings.

We conclude that group-based multicomponent positive psychology intervention programmes, such as the Inspired Life Program, delivered through small-group discussion and activity sessions, could potentially improve the mental health and well-being, build resilience, and increase vocational productivity of individuals residing in rural, resource-limited settings. We urge researchers conducting community-based GBCIs to collaborate with local communities (and their leaders) in their research efforts and to respect and uphold the cultural practices and values of the local population throughout the research process. Given that there is a limited number of mental health professionals in Ghana and sub-Saharan Africa more generally, we found the group-based approach to be cost-effective (since the costs of mental health professionals are distributed across several people, rather than a single individual) and a potentially viable approach to mental health delivery. We contend that a well-organised group-based health behaviour change intervention session could have important policy implications, in that it would offer a cheaper alternative to delivering mental health improvements to rural poor adults in Africa.

## Data Availability

Not applicable.

## Abbreviations

CBPR: Community-based participatory research
GBCIs: Group-based behaviour change interventions
IC: Informed consent
ILP: Inspired life programme
IPA: Innovations for Poverty Action
MRC: Medical Research Councils
mPPI: Multicomponent positive psychology intervention
PMR: Progressive muscle relaxation
